# Apolipoprotein C1: Its Pleiotropic Effects in Lipid Metabolism and Beyond

**DOI:** 10.3390/ijms20235939

**Published:** 2019-11-26

**Authors:** Elena V. Fuior, Anca V. Gafencu

**Affiliations:** Institute of Cellular Biology and Pathology “N. Simionescu”, 050568 Bucharest, Romania; elena.fuior@icbp.ro

**Keywords:** apolipoprotein, apoC1, *APOC1* gene, lipid metabolism, Alzheimer, atherosclerosis, sepsis, polymorphism

## Abstract

Apolipoprotein C1 (apoC1), the smallest of all apolipoproteins, participates in lipid transport and metabolism. In humans, *APOC1* gene is in linkage disequilibrium with *APOE* gene on chromosome 19, a proximity that spurred its investigation. Apolipoprotein C1 associates with triglyceride-rich lipoproteins and HDL and exchanges between lipoprotein classes. These interactions occur via amphipathic helix motifs, as demonstrated by biophysical studies on the wild-type polypeptide and representative mutants. Apolipoprotein C1 acts on lipoprotein receptors by inhibiting binding mediated by apolipoprotein E, and modulating the activities of several enzymes. Thus, apoC1 downregulates lipoprotein lipase, hepatic lipase, phospholipase A2, cholesterylester transfer protein, and activates lecithin-cholesterol acyl transferase. By controlling the plasma levels of lipids, apoC1 relates directly to cardiovascular physiology, but its activity extends beyond, to inflammation and immunity, sepsis, diabetes, cancer, viral infectivity, and—not last—to cognition. Such correlations were established based on studies using transgenic mice, associated in the recent years with GWAS, transcriptomic and proteomic analyses. The presence of a duplicate gene, pseudogene *APOC1P*, stimulated evolutionary studies and more recently, the regulatory properties of the corresponding non-coding RNA are steadily emerging. Nonetheless, this prototypical apolipoprotein is still underexplored and deserves further research for understanding its physiology and exploiting its therapeutic potential.

## 1. Introduction

Apolipoproteins are the physiological agents for the transport along the body of aqueous fluids of the hydrophobic lipids. For this, apolipoproteins take part in the assembly of supramolecular complexes—lipoproteins, which are categorized based on their increasing buoyant density into: chylomicrons, VLDL, IDL, LDL, Lp(a), and HDL [1]. Besides the structural role in the formation of lipoproteins, the apolipoproteins actively participate in the metabolic processing of both endogenous and exogenous lipids, serving as ligands for cell membrane receptors and modulating the activity of relevant enzymes, transporters, and lipid transfer proteins, as reviewed in [2,3]. Some of the apolipoproteins (e.g., apoB) are confined to certain types of lipoproteins, others—the exchangeable ones (e.g., apoA1, C1, C2, C3, and E), are able to transfer between the lipoprotein classes. Interestingly, a number of the genes encoding apolipoproteins are organized into clusters, probably for a more efficient coordinated regulation. In humans, there are two clusters: *A1/C3/A4/A5* located on chromosome 11 [4,5,6], and *E/C1/C4/C2* on chromosome 19 [7], while the murine homologues lie on chromosome 9, respectively 7. Apolipoproteins encoded by the genes in the *E/C1/C4/C2* cluster are important for controlling plasma lipid levels, with subsequent implications in cardiovascular physiology and pathology. ApoE has multiple functions, primarily being involved in the receptor-mediated uptake of plasma lipoproteins and also in the cholesterol efflux from macrophages, which prevents the formation of pro-atherogenic foam cells as reviewed in [8]. In humans, there are three major *APOE* alleles—*APOE2*, *APOE3*, and *APOE4*; in addition, *APOE* genotypes correlate relatively linearly with LDL-cholesterol levels and the coronary risk [9]. At present, ApoC1 is the only known endogenous inhibitor of cholesteryl ester transfer protein [10], and this constitutive action of apoC1 is impaired in coronary artery disease of dyslipidemic patients [11]. ApoC4 participates in triglyceride metabolism [12] and, when overexpressed, provokes hepatic steatosis [13]. ApoC2 functions as a cofactor for lipoprotein lipase (LPL) and its deficiency results in type I hyperlipoproteinemia [14].

Although the three *APOE* alleles appear to be determinant for the plasma lipidemia [15], other apoE-independent polymorphisms found in the cluster contribute to lipid homeostasis [16].

Noteworthy, the gene encoding LDLR, the receptor whose deficiency leads to hypercholesterolemia, is also localized on the chromosome 19, although on the p arm [17].

Extensive research was carried out on apoE, as a result of two major discoveries related to two dreadful pathologies of the modern developed societies: (i) apoE deficient mice (*APOE*^−/−^) spontaneously develop atherosclerosis [18] and (ii) *APOE4* allele is a genetic risk factor for Alzheimer’s disease [19]. Much less was investigated in its neighbor, the apolipoproteinC1 gene (*APOC1*), and its corresponding polypeptide (apoC1); yet a significant part of that research was prompted by the physical proximity of the two genes in the genome.

This association have led in various situations to confounding results, and thus apoC1 continues to remain a relatively elusive issue to our current comprehension. Moreover, the latest dedicated reviews date two decades and they consist of: (i) a comprehensive understanding at that time, systematic comparative analysis of apoC1, apoC2, apoC3, as members of a functional family [20], and (ii) an overview of the results obtained by genetic manipulation of the corresponding genes in mice [21]. 

Herein, we overview the main aspects regarding apoC1 structure and activity with a focus on more recent findings, as well as we underscore some of the less clarified aspects.

## 2. *APOC1* Gene

### 2.1. APOC1 Gene and Pseudogene Localization

Genetic mapping of *APOC1* gene on human chromosome 19 was achieved by studies on hybrid rodent-human cells, and the presence of the clustered genes for apolipoproteins E, C1, C4, and C2 suggested at once that they may be coordinately regulated [7]. *APOC1* gene is located ~5 kb downstream of *APOE* gene in the same orientation [22]. 

At 7.5 kb downstream of *APOC1* gene, and oriented in the same direction, is located the *APOC1P1* pseudogene. This non-coding copy of *APOC1* was originally supposed to have been evolved by duplication, followed by the substitution of the penultimate codon for the signal peptide sequence with a stop codon [23]. Intriguingly, in the case of large primates (bonobo *Pan paniscus*, chimpanzee *Pan troglodytes*, orangutan *Pongo abelii*), in the *APOC1P1* locus there exists a true gene (*APOC1A*), which codes for an acidic isoform. Surprisingly, top-down sequencing indicated that this locus may represent a non-duplicated, distinct gene from *APOC1*, which encodes the apoC1 basic isoform, also designated as apoC1B [24]. Supplementary data are needed to address the precise role of this pseudogene. Recent advances in research demonstrated that long non-coding RNA is endowed with regulatory properties, as reviewed in [25]. In this respect, a recent report identified polymorphism rs5112 in *APOC1P* to significantly affect LDL-cholesterol levels in non-Hispanic Whites [16]. In addition, non-coding RNA APOC1P1-3 was found to reduce α-tubulin acetylation and consequently block apoptosis in breast cancer [26] and to regulate migration and inflammation in cholangiocarcinoma [27]. Further studies may elucidate whether *APOC1P* plays any role in regulating lipid metabolism.

### 2.2. Gene Structure: Encoding and Regulatory Elements

**Figure 1:**
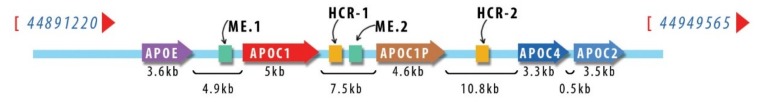
The relative position of human *APOC1* gene in the cluster on the chromosome 19. Schematic representation of the apolipoprotein gene cluster on human chromosome 19 based on data from PubMed (www.ncbi.nlm.nih.gov/gene/341), together with the corresponding regulatory elements; distances were calculated based on sequence NC_000019.10. Abbreviations: apo, apolipoprotein; *APOC1P*, pseudogene; HCR, hepatic control region; kb, kilobase; ME, multienhancer.

Human APOE/C1/C4/C2 cluster is located at position 19q13.32. *APOC1* gene extends 5.0 kb, contains 4 exons, and mRNA translation generates an 83 amino acid precursor [23]. In the cluster, the genes are interspersed with the regulatory elements, which are duplicated, such that HCR (hepatic control region) is present as HCR-1 [28] and HCR-2 [29] and ME (multienhancer) has two copies, ME.1 and ME.2 [30]. The relative position of the apolipoprotein genes and of the regulatory elements in the cluster on the human chromosome 19 is schematically depicted in Figure 1.

Flanking Alu sequences were suggested to be involved in the duplication processes [31]. Mobile genetic elements were also shown to be engaged in *APOC1* transcriptional regulation, as a human endogenous retroviral element (HERV-E element) enhanced the transcription in variable degrees in many tissues, from 15% in the liver up to 50% in the brain from the total amount produced [32]. Of note, the retroviral transcripts were generated by alternative splicing, yet the final polypeptide was identical with that produced from the *APOC1* classical promoter. 

The liver and the macrophages are the two major biosynthetic sites of apoC1 [23]. In the liver, HCR-1 controls predominantly the expression of *APOE* and *APOC1*, while HCR-2 controls mainly the expression of *APOC4* and *APOC2* [33,34], but at least one of them is needed [35].

The nuclear receptor TR4, for which there exists a response element within HCR-1, controls the expression of apoE, apoC1, and apoC2, as demonstrated in HepG2 human hepatocyte line and TR4 knock-out mice [36]. In macrophages, apoC1 production is controlled by multienhancers ME.1 and ME.2 via a liver response element (LRE) to the transcription factor liver X receptor (LXR), which heterodimerizes with retinoid X receptor (RXR) [37]. Furthermore, in macrophages, ME.2 enhances the activity of the promoters of all genes in the cluster, supporting the coordinated regulation of the cluster [38].

The transcriptional regulator PPARγ, which presents a conserved response element in the intergenic region apoE/apoCI [39], is also involved in modulating both apoC1 and apoE expression. Thus, the antidiabetic drug ciglitazone raised the expression of apoE, but reduced that of apoC1 in HepG2 cells, similarly to the effect of the endogenous PPAR-γ ligand, 15d-prostaglandin J_2_ [40]. On the other hand, more recently, it was reported that, in the same cell line, PPAR-γ decreased the expression of both apoE and apoC1, either by using shRNA silencing or pharmacological agonists pioglitazone and rosiglitazone [41].

The promoter of *APOC1* also contains a binding element for Zfp125. This is a FOXO-1 inducible hepatic transcriptional repressor, which reduces lipoprotein assembly and VLDL secretion by acting on no less than 15 lipid-related genes (including *MTTP*, *APOE*, *APOA4*, *APOA1*, *ABCA1*, *LDLR*, *LCAT*, *APOH*, *SCARB1*, *FABP1*,*4*,*5*), thus supporting lipid accumulation and liver steatosis in mice [42]. Additionally, apoC1 expression is modulated by Znf 202, another transcriptional repressor, which controls HDL levels and affects lipid homeostasis by downregulating genes from the clusters *APOE/C1/C4/C2* and *APOA1/C3/A4/A5* [43].

### 2.3. Significant Polymorphisms

In humans, several *APOC1* gene polymorphisms were correlated with altered plasma lipid levels and related dysfunctions. One of the best characterized is the HpaI polymorphism (rs 11568822) (allele H2), generated by the insertion of the CGTT sequence in the position −317 of *APOC1* promoter [44], which leads to an increased level of apoC1, with subsequent type III hyperlipoproteinemia [45] and also raises serum C-reactive protein [46]. Importantly, this polymorphism appears to be associated with apoE and Alzheimer’s disease (AD), as *APOE4* allele represents a risk factor for this pathology. The *APOC1* gene is in linkage disequilibrium with the *APOE* gene and a series of studies indicated the H2 allele of *APOC1* as a genetic risk factor for AD, in association or separately from *APOE*4 allele [47,48,49,50]. However, despite the bewildering number of studies involving specific populations or groups of patients, the association proves significant only when the number of analyzed samples confers the required statistical power [51] and thus, the number of reliable reports reduces dramatically. A meta-analysis performed on 2685 controls and 2092 AD patients indicated that (i) the H2 allele correlated with an increased AD risk in Asians, Caucasians, and Caribbean Hispanics, but not in African Americans, and (ii) this allele was found preferentially in carriers of *APOE4* allele [52]. This indel was also reported to be associated with gallstone disease in women [53].

The G allele of rs4420638 SNP was correlated with increased LDL-cholesterol [54], increased risk of coronary heart disease in both Europeans and Asians [55], and also AD risk [56]. 

Based on sequencing of *APOE/C1/C1PC4/C2* and HCR regions, a recent comprehensive analysis demonstrated that, besides the common SNPs, in two ethnic groups comprising 623 non-Hispanic Whites and 788 African Blacks there were also rare variants that associated significantly and apoE-independently with lipid traits [16]. In addition, the same study showed that the pattern of correlations with LDL-, HDL- and total cholesterol, as well as triglycerides, apoB and apoA-I was influenced by the ethnicity.

Another naturally occurring polymorphism is T45S, which is found with a higher prevalence in the Amerindian population. It was reported that T45S exhibited a stronger preference towards VLDL than to HDL [57] and also in one study, based on 410 Canadian Oji-Cree individuals, where the frequency of the S45 allele was 8%, it was associated with reduced obesity and decreased plasma concentrations of leptin and apoC1 [58]. However, another report based on 228 American Indians, with 15.8% frequency of S45, indicated that T45S was associated with a 9% higher body mass index, and may be related to the increased prevalence of diabetes in the isolated ethnic populations [59]. Further studies on larger groups are definitely needed to elucidate the role of this mutation.

## 3. Protein Expression and Structure

Besides liver and macrophages, smaller amounts of apoC1 protein were detected in the spleen, lungs, skin, brain, and kidney [23]. The protein is synthesized at the endoplasmic reticulum, as an 83 amino acid precursor with a molecular mass of 9.3 kDa. After the co-translational cleavage of the signal peptide MRLFLSLPVLVVVLSIVLEGPAPAQG, the 6.6 kDa mature protein secreted into the extracellular space encompasses only 57 amino acids, being the smallest of all apolipoproteins. With a high content of basic amino acids comprising 9 lysine and 3 arginine residues and an apparent isoelectric point of 6.5 [60], apoC1 is the most positively charged apolipoprotein.

Soon after the elucidation of apoC1 sequence, it became apparent that it contains four 11-residue sequence repeats. Since this motif is also encountered in apolipoproteins such as A1, A2, and C3, the hypothesis of a common ancestor was suggested and an evolutionary tree was proposed accordingly [61]. Thus, bioinformatic analysis of amino acid sequences reinforced gene duplication as the driving force for the emergence of new proteins capable of binding and transporting lipids, while the newly evolved elements were further tuned by gene elongation and point mutations. 

ApoC1 free in solution exhibited a secondary structure with ~30% α-helix, but in the lipoprotein-like environment established by SDS micelles, the α-helix content raised to 54%, according to circular dichroism measurements [62]. The increased ordering was due to a structure with two amphipathic α-helices of class A [63], as evidenced by NMR [62]. 

According to this report, the two α-helices are located in the regions 7–29 and 38–52, separated by a flexible hinge. The N-terminal helix appears more flexible than the C-terminus. The hydrophobic faces of the helices support the contact with the lipids, depending on their flexibility: the C-terminal helix anchors the lipids, while the N-terminal helix helps establish specific contacts with lecithin cholesterol acyltransferase (LCAT), an enzyme activated by apoC1. A series of phospholipids were shown to induce the transition towards an ordered, helical structure of the C-terminal region [64,65]. Depending on the hydrophobic moiety-SDS or phospholipid- used to induce the ordering of the polypeptide, and perhaps due to experimental artefacts, slightly different marginal residues were reported for both the N-terminal [66,67,68] and the C-terminal helix [65,68,69,70,71], but the overall 3D representation remains similar. The two helices of apoC1 mediate the reorganization of lipid bilayers and consequently, the ABCA-1 dependent cholesterol efflux, yet with different kinetics, the C-terminal helix acting fast, and the N-terminal one-slow [72]. Nonetheless, domain swapping in apoC1 did not abolish the efflux, as it occurred in the case of apoA2. This opposite behavior of apoA2, which also displays its two helices oriented similarly, may be explained by the existence of additional structural determinants in apoA2 polypeptide chain consisting of 77 amino residues. To further strengthen the importance of the C-terminal helix of apoC1 in lipid binding, 1,2-dimyristoyl-3-sn-glycero-phosphocholine was shown to protect residues 38-51 from proteolysis [71]. In the same study, single point mutagenesis indicated aromatic residues F42 and F46 to be pivotal for the stability and physiology of apoC1. Thus, their replacement with either Ala or Gly altered both the capacity of interacting with phospholipids and the discoidal morphology of the particles, as demonstrated by sedimentation velocity studies. In fact, these residues, clustered together with W41, were previously predicted to act as a local nucleation center [73].

Despite a certain variability in the primary structure of apoC1, based on the comparative analysis of the protein sequence in various species (man, baboon, dog, rat), the hydropathy plots indicated that the structural domains are conserved [74].

Recently, the X-ray structure revealed that apoC1 forms dimers with a certain degree of flexibility, such that four crystal forms of the dimers were identified, which may impart versatility in exerting the physiological activities [75]. Nonetheless, it was known for about two decades that the self-association of apoC1 is not stable, and other apolipoproteins and/or lipids may participate in the binding and retention to lipoprotein particles [76].

**Figure 2:**
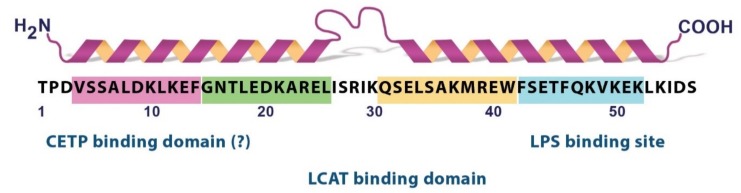
Schematic representation of the principal structural domains of apoC1. Main structural determinants of apoC1 are four 11-mer sequences [61], two helical domains [62,65,66,67,68,69,70,71], LPS binding site [77], lecithin cholesterol acyltransferase (LCAT) binding region [70]. Cholesteryl ester transfer protein (CETP) binding domain is still ambiguous, either in N-terminal or C-terminal domain [78,79].

In Figure 2, we present the main structural determinants of apoC1 as a summary of the aforementioned studies. 

The presence of the 11-mer based amphipathic helix is the underlying molecular basis of the capacity of apoC1, as well as of the other exchangeable proteins, to reversibly adsorb and desorb from the lipoproteins and also to transfer between the various classes [63,80]. In an elegant biophysical study, based on measurements of the interfacial tension upon apoC1 binding to model triglyceride/water and phospholipid/triglyceride/water interfaces, Small’s group brought evidence that variation of the lipoprotein size affects the binding of apoC1, which adsorbs upon expansion and desorbs upon decompression [81]. In addition, this may explain the ability of the various apolipoproteins to replace each other, as a function of their affinities for the lipoprotein particles. Also, with the aid of rational mutagenesis, which designed single Pro or Ala mutations that induce significant alterations of the helical content, it was concluded that peptide-lipid interactions determine binding and retention of the helical polypeptide chains to lipoproteins [82].

Mass spectrometry studies showed that apoC1 was also present in plasma as a polypeptide of 55 amino acid residues, as a result of the proteolytic cleavage of the two N- terminal amino acids by dipeptidyl peptidase IV. Notably, the ratio between the two plasma isoforms seemed to be an important parameter to diagnose mucopolysaccharidoses, associated with increased levels of the enzyme dipeptidyl peptidase IV [83]. 

Moreover, T45S mutant exhibited an increased susceptibility towards proteolysis [57] and this correlates with T45 being an essential residue in the stabilization of the helical structure of apoC1 monomer, as demonstrated by the fully unfolded point mutant T45P [84].

## 4. The Biochemistry of ApoC1: Modulating the Plasma Lipid Profile

ApoC1 is present in chylomicrons, VLDL and HDL, being an exchangeable apolipoprotein between the various lipoprotein classes, with an important role in lipid homeostasis. In fasting conditions, apoC1 is found predominantly in HDL, while post-prandially, it transiently attaches on the surface of triglyceride rich lipoproteins such as chylomicrons and VLDL [85,86]. Although initially the distribution of apoC1 in subfractions HDL_2_ and HDL_3_ was reported to be similar [87], more recently, anion-exchange chromatography led to an advanced fractionation of the heterogeneous HDL class into five categories (H1-H5), apoC1 being exclusively associated with H1 [88]. An average apoC1 plasma concentration of ~60 ± 15 mg/L was reported in normolipidemic humans, while in hyperlipoproteinemia type IIa, IIb, IV, and V values raised to 70 ± 20, then significantly to 100 ± 20, 100 ± 20 and 260 ± 94, respectively [89]. 

**Figure 3:**
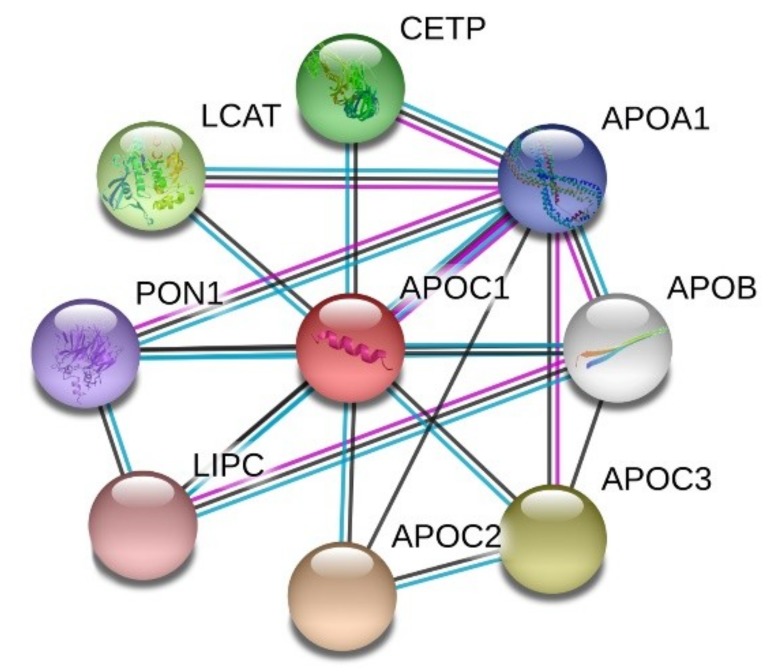
Human apoC1 protein-protein interaction network. STRING analysis (https://string-db.org/) revealed the closest partners, based on evidence: magenta—experimentally determined, black—coexpression and blue—from curated databases. Abbreviations: APO—apolipoprotein; CETP—cholesteryl ester transfer protein; LCAT—lecithin–cholesterol acyltransferase; LIPC—hepatic lipase; PON-1—paraoxonase 1.

STRING analysis of the protein-protein interaction network revealed that apoC1 interacts with other apolipoproteins such as apoA1, B, C2, C3, but also with hepatic lipase (encoded by *LIPC* gene), cholesteryl ester transfer protein (CETP) and enzymes LCAT and paraoxonase (PON1), as shown in Figure 3.

ApoC1 was shown to inhibit apoE-mediated lipoprotein binding to the corresponding receptors. Thus, in vitro, apoC1 inhibited apoE-mediated binding of IDL (intermediate density lipoproteins) and VLDL to both LDL receptor (LDLR) [90] and lipoprotein receptor-related protein (LRP) [91] in cultured fibroblasts. In vivo, human apoC1 overexpression in mice led to hyperlipidemia due to the reduced uptake of VLDL and post-lipolysis particles [92] by inhibiting binding to VLDLR [93]. 

It was also shown that apoC1 inhibited apoA1-mediated uptake of HDL cholesteryl esters by receptor SRB-1 in vitro, and adenoviral-mediated overexpression of apoC1 in mice led to an increased HDL level in vivo [94].

Similarly to other apolipoproteins, apoC1 is a direct modulator of several enzymes involved in lipoprotein metabolism. Thus, apoC1 is an activator of LCAT, an enzyme responsible for converting discoidal, cholesterol-rich HDL into spherical, cholesteryl ester-rich HDL mature particles. ApoC1 activates LCAT in a lesser extent than apoA1, yet with different substrate requirements. Activation by apoC1 occurs regardless of the saturation status of the fatty acyl chains of phosphatidylcholine substrates, whereas apoA1 behaves discriminatorily in this respect [95]. Furthermore, albumin potentiated LCAT activation by apoA1, but inhibited activation by apoC1 [96]. This may suggest that LCAT inhibition by the two apolipoproteins is not a mere redundancy, but rather, they may manifest in different contexts, conferring a subtle LCAT modulation as necessary. Studies with synthetic peptides led to the conclusion that LCAT activation is fully achieved with fragment 17–57, and in particular, the region 32–57 constitutes a major phospholipid-binding determinant of apoC1 [70].

ApoC1 was also shown to affect the activity of lipoprotein lipase (LPL), an enzyme anchored on the surface on the endothelial cells, which hydrolyzes triglycerides and promotes metabolization of chylomicrons and VLDL upon activation by apoC2. Early studies on a rat heart identified an inhibitory effect of apoC1 on apoC2-activated LPL [97]. 

Moreover, in *APOE* knock-out mice, endogenous apoC1 inhibited LPL, depending on its expression level, subsequently increasing VLDL and inducing hyperlipidemia [98]. Noteworthy, in patients with chronic renal failure, who underwent hemodialysis, VLDL-bound apoC1 was depleted, rendering VLDL a better substrate for LPL and improving lipid processing [99]. The mechanism of LPL inhibition by apoC1, as well as by apoC3, involves enzyme displacement from the lipid droplets in a manner depending on apolipoprotein concentration. Interestingly, these apolipoproteins also assist the irreversible inactivation of LPL by angiopoietin like 4 Angptl4 [100], a protein emerging as a powerful anti-atherogenic molecule [101,102].

Hepatic lipase (HL), which promotes conversion of IDL into LDL, was likewise inhibited in vitro by apoC1 [103,104]. Also, apoC1 was reported as an inhibitor of phospholipase A2 [105], a proinflammatory molecule found on LDL. ApoC1 interacted with circulating free fatty acids, probably via the positively charged LPS-binding site, thus preventing their uptake by the cells and the subsequent intracellular esterification [106].

Transgenic mice for human *APOC1* displayed increased plasma levels of cholesterol and triglycerides, correlated with the number of copies of the transgene [106,107]. The severe hypertriglyceridemia manifested independently of apoE, as it also occurred in *APOE* deficient mice [104]. Thus, besides apoE displacement, supplementary mechanisms had to be taken into account, such as inhibition by apoC1 of LPL [108] and HL [104]. 

In humans, HDL-associated apoC1 was identified as a potent inhibitor of CETP, with IC_50_ 100 nM [109]. CETP mediates the exchange of neutral lipids—cholesteryl esters and triglycerides—between lipoproteins, resulting in an increase of VLDL and LDL, while depleting HDL, with an expected pro-atherogenic overall outcome. Thus, CETP inhibition was perceived as a straightforward therapeutic approach against atherosclerosis, but in fact, it turned out to be a more complicated issue due to the genetic variation and pleiotropic actions of CETP [10,110]. 

The structural basis of CETP inhibition by apoC1 relies on electrostatic interactions [111], but it is not clear which region is involved in this activity. Thus, a study on dyslipidemic baboons showed that a naturally occurring peptide, comprising the first 38 amino acids (4 kDa, pI = 7.1), inhibited CETP activity both in vitro and in vivo [79], while another study on human CETP attributed this inhibitory effect to the C-terminal peptide (amino acids 34–54) [78]. The glycosylation of apoC1 also affects its electrostatic properties and, subsequently, the inhibition of CETP, and this could explain, at least partially, the increase of plasma activity of CETP in diabetic patients [112]. Also, in dyslipidemic patients, CETP inhibition by apoC1 was obstructed, a fact attributed to the sequestration of the apoC1 polypeptide on the increased VLDL fraction, which may render it inactive [11]. The physiological significance of inhibiting CETP by apoC1 is not completely understood, since in rabbits apoC1 does not inhibit CETP [113], and mice are naturally deficient in CETP [114]. In vivo, mice transgenic for CETP on an apoC1 knock-out exhibited increased levels of cholesteryl esters in VLDL, as compared with control mice [115]. However, in mice expressing both human CETP and apoC1 [116], despite a reduction in the specific CETP activity, both the mRNA and protein levels of CETP were increased, as a result of the indirect effect of LXR activation by the hyperlipidemic milieu and consequently, the expected lipid profile with increased HDL on the expense of VLDL was not achieved. 

**Figure 4:**
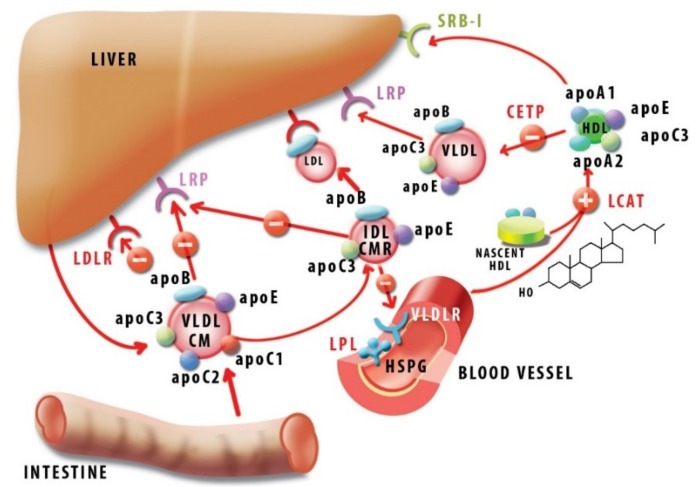
Involvement of apoC1 in lipid homeostasis. The picture schematically represents the main stages of lipid metabolism and its modulation by apoC1 (+: activation, −: inhibition). ApoB was represented in a simplified form regardless of its participation as apoB48 or apoB100. Abbreviations: apo-apolipoprotein; CETP—cholesterylester transfer protein; CM—chylomicrons; CMR—chylomicron remnants; HDL—high-density lipoprotein; HL—hepatic lipase; HSPG—heparan-sulfate proteoglycans; IDL—intermediate density lipoproteins; LCAT—lecithin–cholesterol acyltransferase; LDL- low-density lipoproteins; LDLR—low-density lipoprotein receptor; LRP—LDLR-related protein; SRB-I—scavenger receptor BI; VLDL—very low density lipoprotein; VLDLR—very low density lipoprotein receptor. Chemical formula represents cholesterol.

In Figure 4, we summarized the main pathways of lipid metabolism in which apoC1 participates.

## 5. ApoC1 in pathology

### 5.1. Atherosclerosis

The role of apoC1 in cardiovascular disease remains debatable. On one side, there is increasing evidence, which supports a pro-atherogenic aspect of apoC1. An early step in the evolution of the atherogenic process is the development of lipid-laden macrophages, known as foam cells [117]. ApoC1 expression was induced during monocyte differentiation into macrophages [23] and increased 25-fold upon macrophage treatment with LXR agonist T013017 [37], suggesting that apoC1 may be implicated in the development of foam cells. These results are further supported by a recent high throughput RNA interference study, whereby *APOC1* silencing reduced LDL uptake by monocyte-derived primary human macrophages [118]. 

ApoC1-enriched HDL appeared to mediate also later atherogenic events, such as induction of apoptosis in aortic smooth muscle in vitro by the recruitment of neutral sphingomyelinase, an event that mimics the in vivo rupture of the atherosclerotic plaque [119]. 

Cardiovascular disease was long connected with high cholesterol plasma levels and the therapies were directed accordingly [120]. Less is known about the risk associated with hypertriglyceridemia, but this is emerging as an important, albeit secondary atherogenic pathway [121,122].

In studies performed on normolipidemic, asymptomatic, apparently healthy adult volunteers, the apoC1 content of post-prandial lipoproteins rich in triglycerides was predictive for atherosclerosis, as assessed by the echography of the carotid artery [123,124]. Nonetheless, other studies positively correlated the carotid plaque with apoC1 content in the fasting VLDL particles [125,126].

Raised levels of apoC1 were also detected in individuals with cardiovascular risk, such as men with metabolic syndrome [127,128], or women, either with polycystic ovarian syndrome [129,130] or post-menopausal with increased body mass index [131].

In an immunohistochemical study on advanced human atherosclerotic tissues, both apoE and apoC1 were reported to have an increased expression as compared with the non-atherosclerotic controls, however the distribution in respect with the necrotic core was different, as apoC1 was found within the area, whereas apoE was located around it [132]. ApoC1, as well as apoC2 and apoE were raised in patients with myocardial infarction, as detected by proteomic analysis [133]. Additionally, oxidized isoforms of apoC1, as detected by mass spectrometry, may be also associated with cardiovascular dysfunction [134,135].

Inflammation and atherogenesis are closely interconnected [136,137], and apoC1 was implicated as a key factor in the development of LPS-induced atherosclerosis. Thus, in *APOE* deficient mice, apoC1 noticeably increased lesions area by 60%, and also systemic and vascular inflammation in animals *APOE*−/− *APOC1*+/+ as compared with the control *APOE*−/− *APOC1*−/− mice, by stimulating the macrophage’s response [138]. A high level of apoC1 was detected by transcriptomic profiling in the atherogenic murine model obtained by ablation of the smooth muscle cell marker SM22α, whereby there was also an NF-κB sustained inflammatory response [139]. Recently, it was reported that apoE functions as a checkpoint in controlling the resolution of inflammation by interacting with C1q in the complement activation pathway and subsequently reducing C5 and the inflammatory burden in atherosclerosis and Alzheimer disease [140]. It would be of great interest, taking into account the inhibition exerted by apoC1 on other apoE actions, to assess whether apoC1 interferes in any way with this process.

Lipid lowering therapies, including statins and combined therapies affect apoC1 levels. Thus, a proteomic analysis on a subgroup of the Leipziger LIFE-Heart Study revealed a decrease in apoC1 level upon statin treatment [141]. An important point to stress is that there is no direct correlation between the level of mRNA and the secreted protein, since the latter is post-transcriptionally regulated, in a cholesterol-dependent manner, at least in vitro in HepG2 cells [142]. In THP-1 macrophages, the observed reduction of mRNA correlated with the level of secreted protein upon treatment with atorvastatin, but not with cerivastatin [143]. 

Various GWAS indicated a dependence of the effect of lipid lowering treatments on plasma LDL-C on various polymorphisms of apoC1 such as rs4420638 [144,145] or rs445925 [146]. In addition, polymorphism rs4803770 [15] was associated with a higher risk of cardiovascular disease. The disequilibrium linkage between apoC1 and apoE was also correlated with an increased cardiovascular risk [147].

On the other side, in certain respects, apoC1 may be deemed as anti-atherogenic. Thus could be apoC1 participation in the induction of the cholesterol efflux mediated by the transporter ABCA-1, as demonstrated in vitro in RAW264.7 cells [72] and transfected HeLa cells [148]. In a proteomic study, on plasma isolated from 10 healthy males, apoC1 positively correlated with the cholesterol efflux and anti-oxidant activity [149]. In line with this finding, another proteomic study indicated a significantly decreased amount of apoC1 in the pro-atherogenic HDL from cardiovascular disease patients [150]. However, in vivo, despite the increased macrophage efflux from macrophages in *APOE*−/− *APOC*1+/+ mice, there was a robust +87% increase of the atherosclerotic lesion of the aortic root, as compared with *APOE*−/− *APOC1*−/−, a result that was attributed to the supplementary hyperlipidemia from both triglycerides and cholesterol [151]. 

Interestingly, an integrative system biology analysis, used to evaluate transcriptomic data from 47 microarrays of carotid endarterectomies from the clinical biobank database BiKE, identified an apoC1-centered, “phospholipid efflux” network as an important node in cardiovascular disease [152]. Moreover, there was a gender-biased *APOC1* gene down-regulation in the plaques, with a two-fold decrease in women, as compared with men. However, these data require further validation. 

As already mentioned, apoC1 inhibits hepatic lipase, effectively displacing it from the cell surface, a process wherein HDL particles from hyperlipidemic patients can no longer engage [153], but the pro- or anti- atherogenic status of HL is itself arguable [154].

In conclusion, the role of apoC1 in atherogenesis cannot be thoroughly appreciated, aside from the rest of the participants in lipid metabolism-apolipoproteins, receptors, and enzymes. It appears that atherogenesis, as a multifactorial process, is controlled not by a single determinant, but rather by the balance between various interconnected lipoprotein species and by their specific spatio-temporal distribution. 

### 5.2. Sepsis and Immunity 

As apolipoproteins from different lipoprotein classes ensure protection in septic conditions [155], a cross-talk establishes between lipid metabolism and immunity, wherein apoC1 appears as an important component. An interesting report showed that in systemic post-surgical inflammation, whether accompanied by infection or not, HDL was decreased and depleted in both apoA1 and apoC1, but enriched in apoE [156].

It was shown that apoC1 bound the lipid A/KDO region of LPS—a component of Gram-negative bacteria—via the conserved motif KVKEKLK (amino acids 48–54), and, thus, it provided protection against *Klebsiella pneumoniae*-induced pneumonia in a dose-dependent manner by increasing the early immune response and prevented lethality in mice [77]. Similar results were obtained in humans. Thus, the plasma levels of apoC1 were drastically reduced in patients with advanced sepsis, but were restored to normal levels in survivors, leading to the conclusion that apoC1 level is predictive for the evolution of a severe sepsis [157]. This finding was further supported in the case of the participants in the study Leiden 85-Plus with 561 cases of age over 85 years [158]. Additionally, a study based on 27 septic patients and 23 healthy controls uncovered a proteome related mostly to dysregulated lipid metabolism, consisting of 159 proteins, among which apoC1 was downregulated [159]. In the same context, high levels of plasma apoC1 were positively correlated with the pro-inflammatory response of patients who underwent cardiopulmonary by-pass and presented endotoxemia during reperfusion [160]. 

The investigation of the molecular mechanism through which LPS activated apoC1 in vitro showed that it occurred via CD14/Toll-like receptor 4 signaling, similarly to LBP (LPS-binding protein) and it involved structural determinants in both the N- and C- terminal regions of the polypeptide when assayed in RAW 264.7 macrophages, as well as C57Bl/6 mice [161].

Interestingly, apoC1 was identified among the eight host serum proteins able to detect active tuberculosis in HIV negative, but not HIV positive individuals in a study on 209 subjects from the New York city area [162], but it is not known whether this may be correlated with the reported downregulation of apoC1 in HIV infection [163]. ApoC1 was also the marker with the best sensitivity and specificity for detecting pneumonia in children [164].

Germ-free mice receiving a high fat diet together with *Enterobacter cloacae* B29 from a morbidly obese volunteer donator exhibited increased inflammation and obesity and altered lipid metabolism with decreased apoC1 [165]. Murine hepatic macrophages isolated from control and *Schistosoma mansoni*-infected animals revealed that the helminths-induced both athero-protection and downregulation of apoC1 [166].

A proteomic study of plasma proteins from patients with a kidney transplant revealed that in patients who also received hematopoietic stem cells, apolipoproteins A1, C1, A2, E, and B were increased, as compared with those whose received a simple transplant [167], thus establishing a link between apolipoproteins and immunological tolerance.

### 5.3. Cognitive Processes

As already aforementioned in Section 2.3, apoC1 may constitute a risk factor for Alzheimer’s disease. A study designed to evaluate the correlation of the volume of the hippocampus with the apoE/apoC1 genotype showed that apoC1 genetic polymorphism has a more pronounced effect, as compared with apoE [168]. Subsequently, it was shown that apoC1 is expressed in astrocytes and endothelial cells from various regions of the human hippocampus, both in healthy and AD subjects [169]. Moreover, apoC1 colocalized with beta-amyloid in the senile plaques from the brains of AD patients, and accentuated the neuronal death induced by the soluble oligomers of beta amyloid. To investigate the role of apoC1 in the cognitive function, the same study employed transgenic mice expressing human apoC1 in their brains. These animals presented similar apoE expression as the control group, but diminished learning capacity and memory compared with wild-type animals in specific tests, effects that were attributed to transgene expression. Moreover, apoC1 deficient mice were also impaired in their cognitive capacities, and exhibited increased expression of pro-inflammatory TNF-α despite the lack of glial activation [170]. Furthermore, apoC1 suppressed glial inflammation in a manner dependent on apoE genotype in humans [171]. Compelling evidence for an apoE-independent AD risk provided by apoC1 haplotypes was provided by a recent GWAS study performed on the mainland Chinese GWS cohort; these findings supported by chromatin interaction data indicated a physical interaction between the *APOE* and *APOC1* loci in human brain in both the fetal and adult state [172].

Another study found *APOC1* as an APOE-independent risk factor for AD in connection with oxidative stress [173]. A recent GWAS analysis performed on large numbers of individuals indicated the heterogeneous polygenetic predisposition to AD in the *APOE* region, as there are no less than 30 polymorphisms from genes *BCAM*, *NECTIN2*, *TOMM40*, *APOE*, and *APOC1* in region 19q13.3 [174], as well as associations with other pathologies such as infections, cancer, diabetes [175,176], and epigenetic changes of the regulatory elements within the apolipoprotein cluster [177]. 

### 5.4. Atopic Dermatitis

The transgenic mice that specifically express human apoC1 in the liver and skin not only presented perturbed lipid metabolism manifested by increased plasma levels of cholesterol, triglyceride and fatty acids, they also developed a secondary particularly interesting phenotype, namely atopic dermatitis [178,179]. The pathological cutaneous condition involved atrophy of the sebaceous glands, and epidermal hyperplasia and hyperkeratosis, with subsequent deterioration of the barrier function of the epidermis and abundant hair loss. This condition could be alleviated by treatment with corticosteroids [180] or probiotics that reduce colon inflammation [181], rendering this experimental model useful in the investigation of the mechanisms of initiation and the therapy of atopic dermatitis [182]. 

Nonetheless, apparently unrelated, but subtly connected with the aforementioned studies, apoC1 was identified among the first 10 most abundantly transcribed genes in the *Peromyscus eremicus* mouse adapted to the desert climate, possibly related to the maintenance of a skin wax barrier against water loss [183]. Overexpression of apoC1 in skin occurred in both cases, yet the result on the skin aspect was opposed. Whichever are the additional regulators to explain this dichotomy is a topic to be further explored and exploited for therapeutic purposes.

### 5.5. Viral Infectivity 

A series of studies documented the involvement of the amphipathic helix motifs of apolipoproteins in the assembly and cell entry of viral particles of HCV [184]. It was shown that apoE is indispensable for assembly and cell-cell transmission of the viral particles [185]. However, other reports support by complementary approaches the direct participation of apoC1 and other members of the apolipoprotein family in HCV life cycle, conferring specific infectivity to the viral particles [186]. It was shown that apoC1 takes part in the assembly of HCV viral particles through the C-terminal region, thus playing a role in the viral replication [187]. This behavior is also encountered for other exchangeable apolipoproteins (apoA1, apoA2, apoC2 and apoC3), which embody in their structure amphipathic helices [184], thus representing a potential target for the anti-viral therapy. 

### 5.6. Cancer

An earlier study reported that apoC1 purified from HDL was responsible for the mitogenic effect of HDL on bovine vascular endothelial cells in vitro [188] and, in the recent years, apoC1 emerged as a molecule involved in cancer progression. Thus, apoC1 was demonstrated to promote cell proliferation in prostate cells in vitro, an effect suppressed by its silencing [189]. Moreover, apoC1 was identified by mass spectrometry in hormone-refractory prostate cancer [190]. In colorectal cancer, apoC1 exerted its proliferative activity by MAPK signaling [191] and in pancreatic cells, in vitro it inhibited apoptosis [192].

A few reports indicated the involvement of apoC1 in breast cancer [193,194]. ApoC1 was described as one of the biomarkers from a panel of five most significant proteins with prognostic value in breast cancer, together with C3a, the component of the complement system, transthyretin, apoA1, and truncated apoH [195]. In a phase I marker trial, apoC1 evolved parallel with tumor progression in lung cancer and was validated as a biomarker from a panel of inflammation-related genes, at both mRNA and protein level [196].

Furthermore, apoC1 was also reported as a marker for Wilms tumor [197], gastric adenocarcinoma [198], refractory multiple myeloma [199], hepatitis B-related hepatocellular carcinoma [200].

These novel results open a pathway for the development of new theragnostic opportunities in oncology. 

### 5.7. Diabetes

In diabetes, a series of perturbations in intestinal physiology leads to an altered chylomicron composition, which contributes to the escalation of the risk of developing atherosclerosis [201]. Interestingly, plasma apoC1 concentration was positively correlated with the level of triglycerides, but not that of visceral fat in both type 1 [202] and type 2 [203] diabetes. Moreover, in male patients with metabolic syndrome, a correlation was established between the levels of apoC1 and apoC3 in plasma and the diminution of corporal adiposity [127].

Studies in human *APOC1* transgenic mice indicated that they are protected from atherosclerosis [204]. Yet, in the same experimental model, overexpression of apoC1 led to glomerulosclerosis, suggesting its involvement in diabetic nephropathy [205].

Increased apoC1 levels were detected by proteomic analysis in cases of diabetic nephropathy in patients with type 1 diabetes [206], supporting the results of genetic association studies [207,208]. 

Earlier reports showed that hyperglycemia induced apolipoprotein glycation [209] and this may lead to various consequences. As already mentioned, apoC1 glycation reduced its ability to inhibit CETP and thus in diabetic patients there is an increased CETP activity [112]. 

The 55 amino acid residues truncated isoform of apoC1 is produced by the action of dipeptidyl peptidase-IV, an enzyme inhibited by sitagliptine. Consequently, treatment with this antidiabetic drug leads to significant changes the ratio between the 55- and 57- amino acid isoforms of apoC1 in plasma [210].

Overall, these results demonstrate that apoC1 plays a role in the evolution of diabetes, and further studies in this direction may lead to a better understanding of the connection between lipid and glucose metabolism. 

## 6. Conclusions

Without pretending to be exhaustive, this work aimed to take a snapshot of the multitude of current aspects regarding apoC1 involvement in physiology and pathology. ApoC1 regulates in a complex fashion, the activity of enzymes and receptors involved in the metabolism of VLDL and HDL, and thus the overall effect on the plasma lipid profile is the result of a subtle control resulting from multiple, apparently opposing effects. 

The smallest and the most basic of apolipoproteins, apoC1 employs a recurrent structural motif—the amphipathic helix—to modulate a variety of biological processes. These events may seem apparently unrelated, but they connect at a molecular level directly or indirectly to the transport and/ remodeling of lipids, mostly of cholesterol, as a precursor of steroid hormones involved in various processes. From neurodegenerative diseases to atopic dermatitis, apoC1 remains a remarkable therapeutic target for future studies.

## Abbreviations

apo	apolipoprotein
CETP	cholesteryl ester transfer protein
CM	chylomicrons
CMR	chylomicron remnants
HDL	high-density lipoproteins
HL	hepatic lipase
HSPG	heparan-sulfate proteoglycans
IDL	intermediate density lipoproteins
kb	kilobase
LCAT	lecithin–cholesterol acyltransferase
LDL	low-density lipoprotein
LDLR	low-density lipoprotein receptor
LRP	LDLR-related protein
LPS	lipopolysaccharide
Lp(a)	lipoprotein a
PLaseA2	phospholipase A2
PON-1	paraoxonase-1
SNP	single nucleotide polymorphism
SRB-I	scavenger receptor BI
VLDL	very low-density lipoprotein
VLDLR	very low-density lipoprotein receptor
